# Detecting and mitigating doppelgänger bias in microbiome data: impacts on machine learning and disease classification

**DOI:** 10.1080/19490976.2025.2554196

**Published:** 2025-09-01

**Authors:** Ruwen Zhou, Siu Kin Ng, Joseph J Y Sung, Sunny Hei Wong, Wilson Wen Bin Goh

**Affiliations:** a Lee Kong Chian School of Medicine, Nanyang Technological University, Singapore; b Department of Gastroenterology and Hepatology, Tan Tock Seng Hospital, National Healthcare Group, Singapore; c School of Biological Sciences, Nanyang Technological University, Singapore; d Department of Brain Sciences, Faculty of Medicine, Imperial College London, London, UK

**Keywords:** Microbiome, machine learning, doppelgänger, pre-processing methodology

## Abstract

Highly similar microbiome samples – so-called “doppelgänger pairs” – can distort analysis outcomes, yet are rarely addressed in microbiome studies. Here, we demonstrate that even a small proportion of such pairs (1–10% of samples) can substantially inflate machine learning performance across diverse disease cohorts including colorectal cancer (CRC), inflammatory bowel diseases (IBD), *Clostridioides difficile* infection (CDI), and obesity. Doppelgänger pairs also bias statistical tests and distort microbial network topology. In predictive models, classification accuracy was artificially boosted by 15–30% points across KNN, SVM, and Random Forest classifiers. In association testing, doppelgängers increased false-positive rates and decreased effect size stability; their removal reduced bootstrap variance by up to 28.3%. Moreover, the removal of doppelgängers yielded more stable networks. These effects were consistently observed across 16S, shotgun metagenomic, and simulated datasets. By accounting for highly similar samples, we reduce analytical noise and false discoveries, ultimately enabling more accurate and biologically meaningful microbiome insights.

## Introduction

The human microbiome is a complex and diverse community of microorganisms inhabiting various body sites and has emerged as a crucial factor influencing human health^1,2^. Studies indicate a critical connection between dysbiosis of these microbial populations and a range of diseases, including colorectal cancer (CRC)^3^, inflammatory bowel diseases (IBD)^4^, obesity^5^, and even mental health disorders^6^. While our understanding of the microbiome has grown significantly, many aspects remain unexplored, necessitating further research. This ongoing investigation heavily relies on microbiome profiling, underscoring the critical importance of developing novel preprocessing methods to enhance data quality and analysis.

Microbiome profiling typically involves sequencing of specific genetic markers, or whole metagenome shotgun sequencing for a comprehensive assessment of the microbial community and its functional potential. From the sequencing process, the resulting data is represented as a matrix, with samples and microbial features (e.g., operational taxonomic units [OTUs] or functional genes) denoted as columns and rows, respectively. Matrices are amendable for a variety of analyses, including statistics and machine learning modeling. However, microbiome datasets, even when represented in matrix form, pose unique challenges for analysis due to high-dimensionality, sparsity, and over-dispersion issues^7,8^.

One critical aspect that is often overlooked is the presence of highly correlated or duplicate samples within the same class (e.g., control or case), referred to as “doppelgänger pairs.” These pairs can introduce biases and confound the results of downstream analyses, particularly when applying machine learning techniques for disease classification or biomarker discovery^9,10^. This phenomenon has been previously described and studied on other omics platforms, such as proteomics and transcriptomics, highlighting its relevance across various biological data types. Notably, previous studies have reported that the presence of doppelgänger pairs in datasets can dramatically inflate accuracy metrics, causing random simulations where the expected average accuracy is around 50% to rise to nearly 100%^9^. Doppelgänger pairs represent a form of data leakage, where the model may learn to recognize specific samples rather than underlying patterns. This can result in over-optimistic performance estimates during internal validation.

The phenomena of doppelgänger effects, i.e., how doppelgänger pairs confound analysis, have never been studied in the microbiome. Hence, we propose to 1) identify doppelgänger pairs in microbiome data using standardized protocol^9,10^; 2) assess the impact of doppelgänger pairs on machine learning model performance; 3) evaluate the influence of doppelgänger pairs on downstream analyses, specifically statistical association tests and network analysis; 4) provide recommendations for handling doppelgänger pairs in microbiome data preprocessing to ensure data integrity and enhance the reliability of subsequent analyses.

By addressing doppelgänger effects, we aim to contribute toward the development of more robust and reproducible methods for microbiome data analysis, ultimately advancing our understanding of the microbiome’s role in human health and disease.

## Methods

### Identification of doppelgänger pairs

Doppelgänger pairs were identified via the published protocol of Wang *et al*^10^. This process involved first calculating the Pairwise Pearson’s Correlation Coefficients (PPCC) between samples. Sample pairs were then grouped based on class labels, e.g., case and control. A correlation cutoff derived from the maximum correlation observed between any case and control sample pair was established. Doppelgänger pairs were identified based on this cutoff. Specifically, sample pairs originating from within the same class, i.e., case–case and control–control, with correlations that exceeded the established cutoff were classified as doppelgängers. This method systematically pinpointed highly correlated samples within each class. We also used the software implementation and tutorial to ensure fidelity to original process (https://doi.org/10.5281/zenodo.7080539)^11^.

To quantify pairwise similarity between samples, we evaluated three correlation measures: Pearson’s, Spearman’s, and Kendall’s correlation coefficients. While Pearson’s correlation was selected as the primary method for doppelgänger identification due to its sensitivity to linear similarity, we also computed Spearman’s and Kendall’s correlations to ensure robustness across different assumptions. All correlation matrices were computed using base R functions, with rank-based correlations implemented for comparative assessment (Supplementary box 1).

The maximum cutoff approach uses the highest correlation value observed among between-class pairs as the threshold for identifying doppelg**ä**ngers. This conservative strategy ensures that any within-class correlation exceeding this threshold represents a degree of similarity that is biologically impossible to achieve between different disease states. We validated this approach using the Wilcoxon rank-sum test to assess distributional differences between between-class and within-class correlation groups, and visualized the separation using histograms to demonstrate the biological rationale underlying the cutoff selection.

Alpha and beta diversity were also conducted to identify functional and biological characteristics differentiating the doppelgänger group and non-doppelgänger group. Bray–Curtis distance was used to compute beta diversity between doppelgänger and non-doppelgänger samples in the CDI cohort. Relative abundance transformation was performed, where raw counts were normalized to proportions. We used permutational multivariate analysis of variance (PERMANOVA) to compare the variance between groups in complex multivariate data. Here, PERMANOVA tests for statistically significant difference in species composition between the doppelgänger and non-doppelgänger groups.

### Population

We observed recurrent instances of doppelgänger pairs across various digestive diseases. These include CRC^12,13^, IBD^14,15^, *Clostridioides difficile* infection (CDI)^16^, and obesity^17^. These diseases were chosen due to their established association with alterations in the gut microbiome. Our selection spanned a spectrum of digestive diseases, from those with a direct, well-characterized connection to gut microbial composition, such as IBD and CDI, to conditions like CRC and obesity, where microbial links are significant but less direct. By including diseases both directly and indirectly related to gut microbiome changes, we aimed to provide a comprehensive view of microbial dynamics across various digestive health scenarios. This allowed for explorations of established microbiome-disease associations and offered insights into the nuanced roles of gut microbes in complex conditions such as CRC and obesity. The diversity of diseases selected in this study enhances the generalizability of our findings, thus contributing to a deeper understanding of the gut microbiome’s role in digestive health and disease.

Our datasets were extracted from the comprehensive MicrobiomeHD database^18^. Our selection criteria prioritized studies with the largest sample sizes. Notably, for IBD and CRC, we expanded our analysis to include studies with smaller sample sizes to ensure comprehensive coverage. Additionally, we generated simulated datasets to further examine IBD-specific microbiome patterns.

To evaluate the generalizability of our doppelgänger identification method to shotgun metagenomic datasets, we applied it to publicly available data from the Inflammatory Bowel Disease Multi-omics Database (IBDMDB, also known as HMP2; http://ibdmdb.org), comprising 1,638 stool metagenomic samples from patients with IBD and non-IBD controls.

### Study design on machine learning models

**Figure 1.**
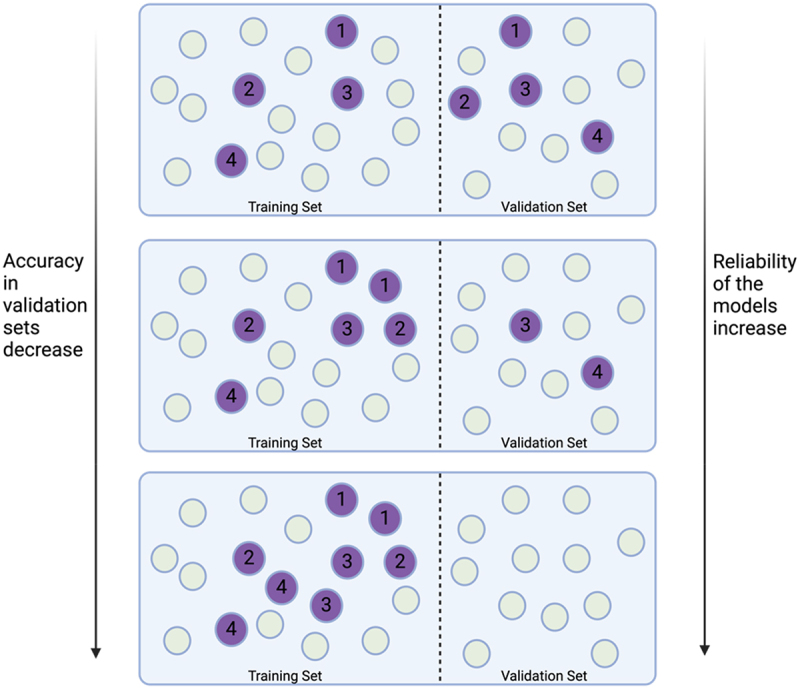
Conceptual graph showing training and validation sets split while having different number of doppelgänger pairs, indicated by identical numerical numbers. The purple circles are doppelg**ä**nger pairs while the rest are normal samples. As we avoid splitting doppelgänger pairs across both training and validation sets, although the accuracy of validation set may decrease, the false positive of the results decreases and the reliability of the evaluation of model performance increases.

The study design assumed that a well-generalized machine learning model exhibits robust performance across various train-test splits. However, even with balanced train-test splits, without investigating the pairwise correlations of samples, the doppelgänger effect could significantly impact the model’s accuracy. Specifically, when highly correlated sample pairs existed in both training and validation sets, they inflate model performance in a dosage-dependent manner^9^. To illustrate this, we use a schematic: In the baseline scenario, all doppelgänger pairs were placed within the training set, thus eliminating expected doppelgänger effects in the validation set. Progressively, as shown in Figure 1, we increased the number of doppelgänger pairs split between the training and validation sets.

In this study, doppelgänger pairs were systematically incorporated into the training and validation sets using a K-nearest neighbors (KNN)^19^ approach, with default of k = 5 to evaluate their impact on model accuracy. KNN, a simple non-parametric supervised learning algorithm, classifies new data points based on the majority class of their “k” nearest neighbors in the training dataset. It assumes that similar data points are close to each other in feature space, making it effective for classification and regression tasks with well-separated data. KNN was selected due to its sensitivity to sample point proximity, effectively illustrating accuracy inflation from doppelgänger pairs. Therefore, with doppelgänger pairs present, KNN predictions could become overly optimistic, as the model “remembered” these pairs instead of learning generalizable patterns.

While our initial implementation focused on the KNN algorithm, we extended our evaluation to include support vector machines (SVM) and random forest classifiers to ensure that the observed effects were not model-specific. KNN was chosen initially for its strong sensitivity to local sample similarity, which makes it particularly prone to performance inflation from highly similar sample pairs. To more rigorously assess model performance beyond simple accuracy, we incorporated additional evaluation metrics including the F1 score and the area under the receiver operating characteristic curve (AUC). These metrics provide a more nuanced assessment of classification quality, particularly under class imbalance or when detecting subtle patterns in microbiome data.

### Study design on network analysis and association test

Since the IBD data has the highest occurrence of doppelgänger pairs, we focus on studying this cohort to evaluate the severity of doppelgänger effects and its downstream impact on association test and network analysis^14^. For the association test, the Wilcoxon rank-sum test was employed to compute the statistical significance of each OTU’s correlation with the disease. The primary objective was to deepen the understanding of the dynamics in microbiome downstream analysis after removing potentially influential sample pairs. In the network analysis, visualization techniques were used to transform microbiome data into a network structure, where each node represented an individual microbe, and edges indicated correlations exceeding a threshold of 0.7. Subsequently, the network underwent an “attack,” where nodes were randomly removed one at a time and this process was repeated iteratively until no nodes remained. Global network attack simulation was conducted to evaluate changes in network characteristics before and after removing doppelgänger pairs by measuring the average path length and average clustering coefficient. The average path length measures the average shortest path between all pairs of nodes in the network. The average clustering coefficient was used to measure the likelihood of a node’s neighbors being interconnected, indicating network fragility. We compare association and network results before and after removal of doppelgänger pairs.

### Study design on assessing different filtering thresholds

We assessed the impact of various filtering thresholds on the identification of doppelgänger pairs in microbiome data. Our filtering strategies were: 1) filtering based on total counts, involving the removal of OTUs with low library sizes; 2) filtering based on prevalence, designed to remove rare OTUs absent in a significant number of subjects; and 3) filtering based on relative abundance, excluding OTUs whose maximum relative abundance exceeded a specified threshold in the samples.

### Bootstrapping for stability assessment

To evaluate the stability of differential abundance estimates, we implemented a bootstrapping framework comparing datasets with and without doppelgänger pairs. For each condition, we performed 100 bootstrap samples by selecting individuals with replacement while maintaining class labels (e.g., case vs. control). Within each bootstrap replicate, we computed the log-fold change (LFC) for each microbial taxon between the two groups. The variance of the LFC estimates across bootstraps was then calculated to assess the robustness of association results.

This bootstrapping approach was applied to both 16S rRNA and metagenomic datasets. It provided a statistical means to quantify the impact of doppelgänger removal on the consistency of effect size estimates, independent of downstream model performance or biological assumptions.

## Results

### Doppelgänger pairs are more prevalent in IBD and CDI datasets

**Figure 2.**
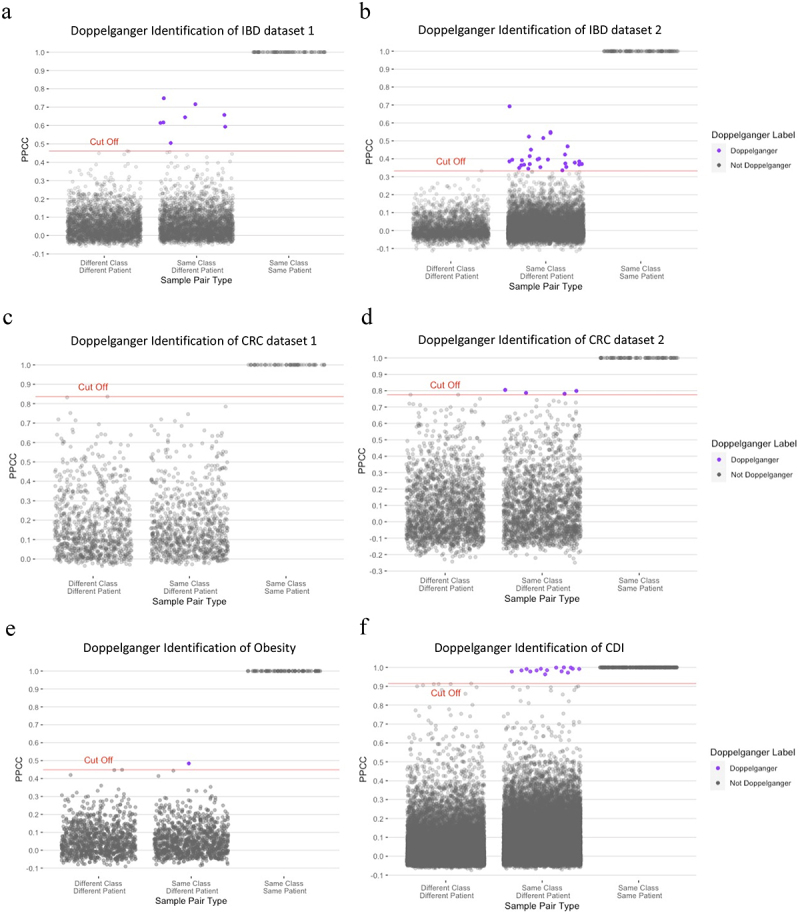
Doppelgänger pairs identified in various microbiome datasets: (a) IBD dataset 1 (8 pairs out of 112 samples), (b) IBD dataset 2 (27 pairs out of 1041 samples), (c) CRC dataset 1 (0 pair out of 44 samples), (d) CRC dataset 2 (4 pairs out of 60 samples), (e) Obesity dataset (1 pair out of 63 samples), and (f) CDI dataset (16 pairs out of 337 samples). Each plot shows the pairwise Pearson’s correlation coefficients between samples, with doppelgänger pairs (correlation above the defined threshold) represented by purple dots and non-doppelgänger pairs by gray dots. The dashed lines indicate the correlation threshold for identifying doppelgänger pairs, which is determined by the maximum correlation between case and control samples. The number of identified doppelgänger pairs varies across datasets, with IBD and CDI datasets exhibiting a higher prevalence compared to CRC and obesity datasets.

**Figure 3.**
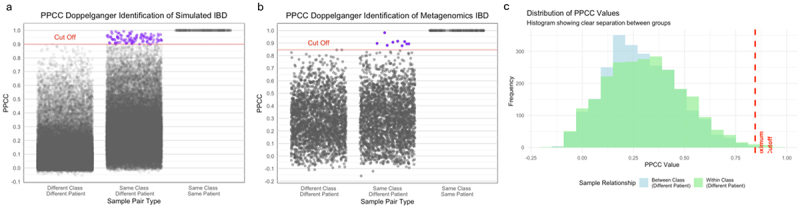
(a) Pairwise Pearson correlation box plot shows identified doppelgänger pairs in a simulated IBD dataset. (b) Pairwise Pearson correlation box plot shows identified doppelgänger pairs in a shotgun metagenomic IBD dataset. (c) Histogram showing the distribution of Pearson correlation coefficients between samples from different classes (between-group, gray) and from the same class (within-group, purple). The vertical dashed line indicates the maximum correlation observed in the between-group distribution.

Doppelgänger identification across different phenotypes revealed that up to 2–10% of samples in IBD and CDI datasets were identified as doppelgänger pairs, whereas only 2–4 doppelgänger pairs were identified – approximately 1% in the CRC and obesity datasets (Figure 2). Consistent with this pattern, analysis of a shotgun metagenomics IBD dataset (IBDMDB) also revealed a substantial number of highly similar sample pairs (Figure 3(b)), reinforcing that doppelgängers are not exclusive to 16S data. Additionally, we applied the method to a simulated IBD dataset and successfully detected injected doppelgänger pairs (correlation > 0.99), further demonstrating that our approach is compatible with both real and simulated microbiome data (Figure 3(a)). Together, these results confirm that our identification method is applicable across 16S amplicon data, shotgun metagenomic data, and synthetic datasets, supporting its generalizability and robustness in diverse microbiome contexts.

The maximum cutoff validation analysis strongly supported the conservative threshold approach for doppelg**ä**nger identification. The Wilcoxon rank-sum test revealed highly significant differences between the distributions of between-class and within-class correlations (*p* = 5.36 × 10^−4^), confirming that samples from the same disease state exhibit systematically higher correlations than samples from different disease states. The histogram distribution clearly demonstrated a natural separation point at the maximum cutoff, where the between-class distribution terminates abruptly while the within-class distribution extends into higher correlation values (Figure 3(c)). This approach achieved perfect specificity by ensuring that no between-class pairs were misclassified as doppelg**ä**ngers, while identifying only the most extreme cases of within-class similarity that represent genuine biological anomalies warranting further investigation.

**Figure 4.**
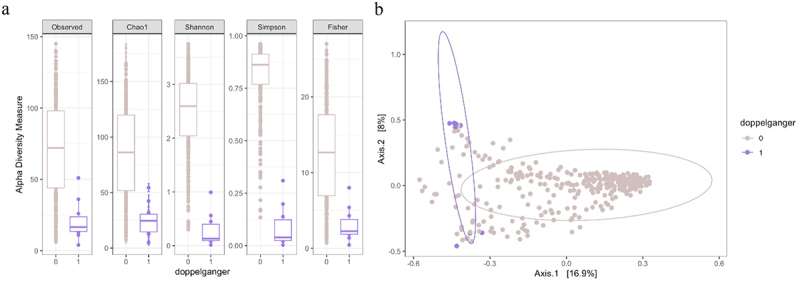
a. Alpha-diversity of samples been identified as doppelgänger pairs in CDI cohort and non-doppelgänger samples; b. Beta-diversity between doppelgänger and non-doppelgänger samples in CDI cohort. Bray–Curtis distance was used to compute beta diversity between doppelgänger and non-doppelgänger samples in the CDI cohort. Relative abundance transformation was performed, where raw counts were normalized to proportions.

We conducted further analysis to identify characteristics and patterns within the doppelgänger group. Figure 4(a) illustrates the Alpha diversity analysis, which indicated that the non-doppelgänger group exhibits greater species richness and evenness. Figure 4(b) illustrates the Beta diversity analysis that showed more variation in species composition within the non-doppelgänger group samples. PERMANOVA yielded a statistically significant result with a p-value of 0.006. This indicates a statistically significant difference in species composition between the doppelgänger and non-doppelgänger groups. These analyses revealed that the doppelgänger group has a markedly different species composition from the non-doppelgänger group, suggesting that the former may suffer from reduced diversity in gut microbiome communities.

### Doppelgängers induce data leakage and inflating accuracy

**Figure 5.**
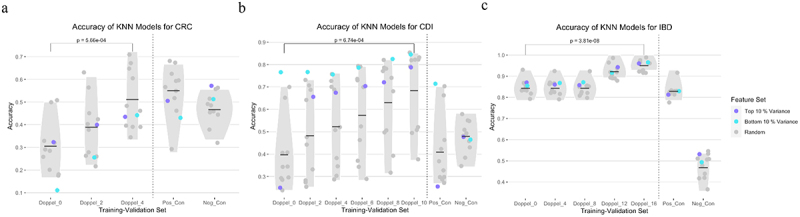
KNN model directly predicts (a) CRC, (b) CDI, and (c) IBD based on gut microbiome. “Doppel_0” represents the baseline accuracy with all doppelgängers in the training set, whereas “Doppel_x” depicts scenarios with an increasing number of doppelgänger pairs shared between the training and validation sets. The negative control was conceptualized as accuracies derived from a binomial distribution, with parameters N (the number of validation samples) and P (the probability of a correct guess, set at 0.5). This simulation represents a random guessing scenario. Conversely, the positive control uses a validation set containing the maximum number of duplicates from the training set.

The impact of doppelgängers pairs on model performance was assessed across multiple datasets using KNN models for CRC, CDI, and IBD. Overall, model accuracies increase with the number of doppelgänger pairs, demonstrating the impact of inflation (Figure 5). These inflated accuracies have little to do with the learning of useful signal from data, and thus. In addition to accuracy, F1 scores and AUC values also increased when doppelgänger pairs were present in both training and validation sets, supporting the hypothesis of performance inflation (Supplementary Figure S1a).

As we increase the number of doppelgänger pairs in the training and validation sets, the accuracy of the model sharply increases, indicating significant distortion due to data leakage. The accuracy of machine learning model inflates from average around 30% to 50% in CRC dataset, from 40% to 70% in CDI dataset and from 85% to 95% in IBD dataset. This trend of inflated accuracy with doppelgänger pairs was consistently observed across KNN, SVM, and random forest models (Suplementary Figure S1b). Even with only a small proportion of doppelgänger pairs present in both the training and validation sets, the performance of the same model on the same dataset becomes unstable. These findings highlight the critical need for rigorous data partitioning to prevent overestimation of model accuracy.

### Improved effect size stability across bootstraps after doppelgänger removal

To further evaluate the effect of doppelgänger removal on the stability of association estimates, we performed bootstrap analysis on both 16S and metagenomic datasets. For each dataset, we computed log-fold changes (LFCs) between case and control groups across 100 bootstrap replicates, and assessed the variance in these estimates as a measure of stability. In the CRC dataset, removing four identified doppelgänger pairs led to an average variance reduction of approximately 9.5% across taxa. In the IBD metagenomic dataset, where nine highly correlated sample pairs were removed, the average variance reduction was approximately 28.3%. These reductions suggest that doppelgänger removal improves the consistency of effect size estimation across resampled subsets, enhancing the robustness of association testing. To supplement this, we defined a small set of highly correlated sample pairs (Pearson’s *r* > 0.995) as pseudo – true doppelgängers, which resemble near duplicates. Our method successfully identified these pairs, supporting its ability to detect biologically implausible redundancies. While we do not claim the discovery of true associations, these findings support the increased reliability and stability of downstream statistical inference after doppelgänger removal.

### Enhanced reliability and biological interpretability of association tests after doppelgänger removal

**Figure 6.**
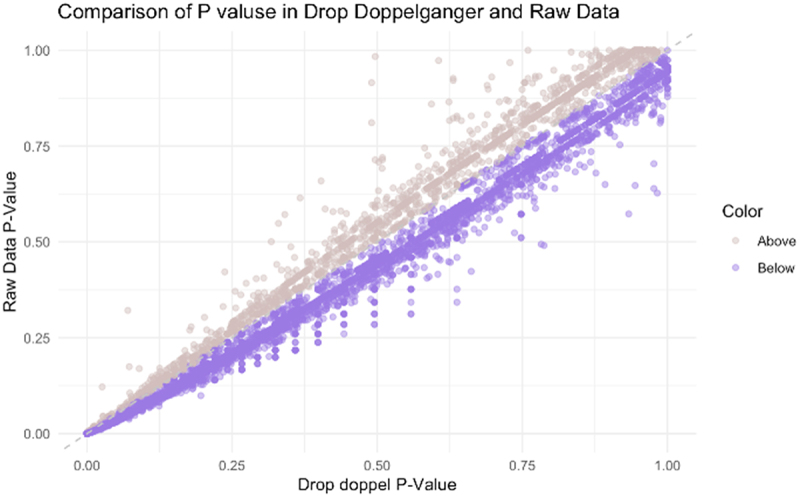
Comparison of p-values for OTU – disease associations before and after doppelgänger removal in the colorectal cancer dataset. Each dot represents an OTU; purple dots indicate higher p-values (less significant) after removal, and pink dots indicate lower p-values (more significant).

We evaluated the impact of doppelgänger removal on the overall pattern of OTU-disease associations using the Wilcoxon rank-sum test applied to each OTU individually. Figure 6 demonstrates that a larger number of OTUs exhibited increased p-values following doppelgänger removal compared to those with decreased p-values. In total, 5,961 OTUs had higher p-values, while 3,573 OTUs had lower p-values, indicating a shift toward more conservative significance estimates after redundancy was addressed. This overall shift suggests that the presence of highly correlated sample pairs can inflate statistical significance by reducing variability within groups. By removing these doppelgänger samples, we mitigate such artifacts, improving the robustness of association results.

Beyond this statistical shift, we also observed a notable enhancement in biological interpretability. Specifically, in the colorectal cancer (CRC) dataset, removal of doppelgänger samples revealed additional genera with literature support. *Blautia*, a well-established short-chain fatty acid – producing genus known to be depleted in CRC, emerged as significantly depleted only after doppelgänger filtering^20,21^. Similarly, *Bilophila*, a pro-inflammatory genus often enriched in CRC, became significantly enriched post-removal^22,23^ (Supplementary figure S2). These taxa were not identified in the full dataset, suggesting that doppelgänger-induced redundancy may have masked real biological signals. The emergence of these canonical CRC-associated genera underscores the value of our approach not only for improving statistical robustness but also for enhancing biological validity.

Our interpretation is that doppelgänger removal reduces noise and the risk of false-positive findings while revealing biologically plausible signatures. These findings emphasize the importance of rigorous quality control in microbiome studies, particularly in high-dimensional association testing where sample redundancy can distort both statistical and biological conclusions.

### Removing doppelgängers alters network topology and structural resilience

We conducted a global network perturbation simulation to evaluate how the removal of doppelgänger pairs affects the structural properties of microbiome co-occurrence networks. Specifically, we assessed two topological metrics: average path length and average clustering coefficient. These metrics serve complementary roles – the former reflects global connectivity and resilience under node removal, while the latter captures local cohesiveness within subnetworks.

**Figure 7.**
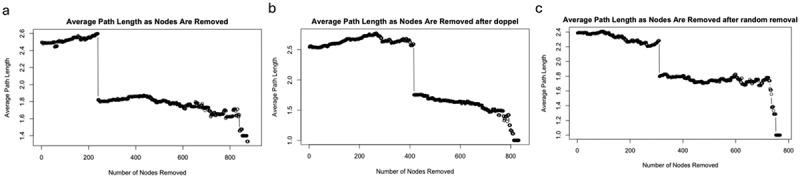
The changes of average path length as nodes are removed for raw data (a), data without doppelgänger pairs (b), and randomly removed samples (c). The average path length measures the average shortest path between all pairs of nodes in the network. As nodes are removed, the average path length initially remains stable but then drops off sharply at a certain point, indicating the disintegration of the network structure. The data without doppelgänger pairs (b) maintains a longer average path length compared to the raw data (a) and randomly removed samples (c), suggesting increased robustness of the network after removing highly similar sample pairs.

**Figure 8.**
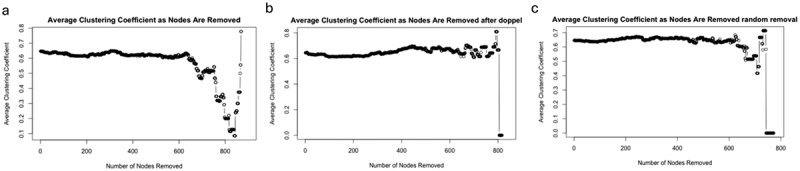
The changes of average clustering coefficient as nodes are removed for raw data (a), data without doppelgänger pairs (b), and randomly removed samples (c). The average clustering coefficient measures the tendency of nodes in a network to cluster together. As nodes are removed, the average clustering coefficient decreases, indicating a reduction in network cohesiveness. The data without doppelgänger pairs (b) shows a more gradual decline compared to the raw data (a) and randomly removed samples (c), suggesting that removing highly similar sample pairs helps maintain the overall clustering structure of the network.

As shown in Figure 7, we measured the average path length of the largest connected component as nodes were iteratively removed. The network constructed from doppelgänger-removed data exhibited a delayed drop-off in average path length compared to both the original and randomly perturbed networks. This suggests that more nodes must be removed before the network undergoes structural fragmentation, indicating greater topological robustness. In contrast, the original network with redundant samples fragmented more quickly, likely due to inflated correlations among doppelgänger pairs. In Figure 8, we evaluated changes in the average clustering coefficient during node removal. Although clustering coefficients naturally decline with node loss, we observed a more gradual decrease in the network without doppelgänger pairs. This trend indicates that redundant samples may artificially elevate local clustering, and their removal promotes a more balanced and interpretable network structure.

These findings suggest improved topological stability after doppelgänger removal, it is less biased by sample-level redundancy, and potentially more amenable to downstream biological interpretation.

### Identification of doppelgängers unaffected by different preprocessing thresholds

In examining the effects of various preprocessing thresholds on doppelgänger identification in obesity and CDI datasets, our results indicate that the filtering process did not significantly alter the number of doppelgänger pairs identified (Table 1). The consistency of identified pairs before and after applying these thresholds underscores the robustness of our method. This novel approach demonstrates stability and reliability across various preprocessing steps, confirming its effectiveness in identifying highly similar pairs regardless of initial data manipulation.

**Table 1.** Number of doppelgängers after conducting filtering with different threshold.

Obesity			CDI		
**Filtering based on total counts:**					
Filtering	Number of doppelgänger pairs found	whether is the same pairs	Filtering	Number of doppelgänger pairs found	whether is the same pairs
library size not equal to zero	1	yes	library size not equal to zero	23	yes
library size larger than 2	1	yes	library size larger than 2	23	yes
library size larger than 5	1	yes	library size larger than 5	23	yes
library size larger than 10	1	yes	library size larger than 10	22	yes
library size larger than 20	1	yes	library size larger than 20	23	yes
library size larger than 50	1	yes	library size larger than 50	23	yes
**Filtering based on prevalence:**					
Filtering	Number of doppelgänger pairs found	whether is the same pairs	Filtering	Number of doppelgänger pairs found	whether is the same pairs
OTUs that are present in at least 2% of the samples	1	yes	OTUs that are present in at least 0.1% of the samples	23	yes
OTUs that are present in at least 4% of the samples	1	yes	OTUs that are present in at least 0.2% of the samples	23	yes
OTUs that are present in at least 6% of the samples	1	yes	OTUs that are present in at least 0.3% of the samples	23	yes
OTUs that are present in at least 8% of the samples	1	yes	OTUs that are present in at least 0.5% of the samples	23	yes
OTUs that are present in at least 10% of the samples	1	yes	OTUs that are present in at least 1% of the samples	31	

## Discussion

As machine learning continues to grow in its application to microbiome data analysis, the integrity and representativeness of the datasets used for training and evaluation become increasingly critical. This study introduced a novel approach to enhance data quality by identifying and mitigating a specific form of data leakage: the presence of highly correlated sample pairs, referred to as doppelgänger pairs. These pairs present two major concerns when inadvertently split between training and validation datasets.

First, the redundancy of information inherent in doppelgänger pairs compromises sample independence and artificially inflates the apparent size and diversity of the dataset. This can lead models to “memorize” rather than generalize, distorting the learning process. Second, and more crucially, when doppelgänger pairs appear in both training and validation sets, they violate the assumption of independence between these partitions, resulting in inflated performance metrics that do not reflect true predictive capability. In other words, the presence of doppelgängers causes information leakage and overestimates model accuracy, ultimately threatening the reproducibility of results. We developed a data-driven approach to identify these pairs using Pearson’s correlation coefficient with a relative cutoff derived from inter-group similarity. This method proved effective across various datasets and data types, including 16S rRNA gene sequencing, shotgun metagenomics (e.g., IBD datasets from HMP2), and simulated microbiome data. We observed that doppelgänger pairs are more prevalent in disease states like IBD and CDI, where inflammation or treatment may drive microbiome convergence among patients, compared to CRC or obesity, where microbial alterations are more heterogeneous.

To evaluate the effects of doppelgänger presence on predictive modeling, we initially used a K-nearest neighbors (KNN) classifier, given its sensitivity to sample proximity. We showed that as the number of doppelgänger pairs split between training and validation sets increased, model accuracy was significantly inflated. To ensure our findings were not model-specific, we extended our analysis to include SVM and random forests. Across all models, we observed consistent patterns of inflation, confirming that the phenomenon is not algorithm-dependent. Moreover, instead of relying solely on accuracy, we evaluated model performance using additional metrics such as F1 score and area under the ROC curve (AUC), which confirmed the same inflation trend. These results underscore the importance of careful data partitioning and quality control in microbiome machine learning studies. Importantly, our method is designed to augment rather than replace standard cross-validation. While cross-validation aims to provide robust performance estimates by rotating training/validation partitions, it assumes independence between samples. By removing doppelgänger pairs before cross-validation, we preserve this foundational assumption and improve the trustworthiness of downstream evaluations.

Beyond predictive modeling, we found that doppelgänger presence also affects biological inference. Association tests conducted on data with doppelgänger pairs showed a greater number of statistically significant results. However, when these pairs were removed, p-values for most OTUs increased, and the number of significant associations dropped. We interpreted this as a reduction in false positives rather than loss of signal. In fact, after doppelgänger removal, several biologically relevant genera emerged that were previously undetected, including *Blautia* and *Bilophila*. These findings suggest that eliminating sample redundancy can uncover more biologically meaningful associations that may otherwise be obscured by spurious signals.

To further explore the stability of association results, we implemented a bootstrapping approach. We found that the variance in log-fold changes decreased by approximately 10–20% after removing doppelgänger pairs, indicating improved consistency of estimated effects. While we do not claim to identify “true” associations, this reduction in variability suggests enhanced reliability and robustness of statistical inference following doppelgänger removal. The increased stability is a desirable property that supports the use of doppelgänger detection as a quality control step in association testing. Additionally, we examined the impact on microbial co-occurrence networks. Network robustness, measured via average path length and clustering coefficient, improved after doppelgänger removal. These changes reflect enhanced topological stability, which may be important for downstream ecological or systems biology analyses. While we acknowledge that structural stability does not guarantee functional or biological relevance, it reduces the likelihood that results are driven by redundant or biased sampling.

Limitations of our study include the potential loss of true biological signals when removing doppelgänger pairs. While our method is conservative by design, future work could explore more nuanced strategies, such as weighting highly similar pairs or incorporating them into model regularization rather than outright removal. Additionally, applying our method to multi-omics datasets could provide deeper insights into the functional consequences of doppelgänger-driven redundancy.

In conclusion, our study highlights a previously underappreciated source of bias in microbiome machine learning and presents a generalizable strategy for improving data quality. By incorporating doppelgänger identification and removal into the preprocessing pipeline, researchers can enhance both predictive validity and biological interpretability of their models. As the field advances, addressing subtle data artifacts like doppelgänger effects will be essential for building reproducible and trustworthy microbiome-based tools.

## Conclusion

This is the first study on doppelgänger effects in microbiome data analysis. We find that accounting for data doppelgängers during the preprocessing stages of microbiome data analysis is essential and overlooked. Specifically, we recommend incorporating doppelgänger pair identification as a quality control step at the conclusion of the preprocessing pipeline in microbiome data analysis. This leads to less inflated machine learning modeling outcomes, and ultimately, toward better insights in how the microbiome contributes or is affected by diseases.

## Supplementary Material

Supplementary_0811.docx

## Data Availability

The source of the datasets used was the comprehensive MicrobiomeHD database: https://zenodo.org/records/569601 and Inflammatory Bowel Disease Multi-omics Database (IBDMDB, also known as HMP2) https://ibdmdb.org. Each dataset has been cited on the manuscript.

## Abbreviations

CDI: Clostridioides difficile infection
CRC: Colorectal cancer
IBD: Inflammatory bowel diseases
KNN: K-nearest neighbors
OTUs: Operational taxonomic units
PPCC: Pairwise Pearson’s Correlation Coefficient
PERMANOVA: Permutational multivariate analysis of variance
SVM: Support vector machines
RF: Random forests
