# Supplementary material for: Detecting and mitigating doppelgänger bias in microbiome data: impacts on machine learning and disease classification
Source: Gut Microbes. 2025 Sep 1;17(1):2554196. doi: 10.1080/19490976.2025.2554196 (PMC12407587; doi:10.1080/19490976.2025.2554196)
Supplement: Supplementary_0811.docx [file KGMI_A_2554196_SM8151.docx]

**Supplementary**

**Correlation method validation**

We experimented with Pearson’s, Spearman’s, and Kendall’s correlation coefficients, observing differences in their distributions, yet found that the relative cut-offs for identifying doppelgänger pairs remain consistent across these methods. The choice of Pearson correlation is rooted in its specific characteristics and assumptions. Given the nature of microbiome data, which may not always adhere to normality or linearity, filtering and normalization must be conducted before the computation of correlation. Log transformation can help in normalizing the distribution, reducing skewness, and making it more symmetrical.

Box 1. Different Correlation Coefficient Methods

Pearson correlation, a parametric measure, assesses the linear relationship between two continuous variables. Its formula is given by:

$$r= \frac{\sum(X_{i}-\overline{X})(Y_{i}-\overline{Y})}{\sqrt{\sum\left( X_{i}-\overline{X} \right)^{2}\left( Y_{i}-\overline{Y} \right)^{2}}}$$

Where $X_{i}$ and $Y_{i}$ are the individual sample points and $\overline{X}$ and $\overline{Y}$ are the means of the samples.

Spearman's rank correlation assesses monotonic relationships (either increasing or decreasing but not necessarily linear) between two variables, calculated as:

$$\rho=1- \frac{6\sum d_{i}^{2}}{n(n^{2}-1)}$$

Where $d_{i}$ is the difference in ranks of each pair of features and n is the number of features.

Kendall's tau correlation measures the strength of association based on the ranks of the data, computed as:

$$\tau= \frac{2}{n(n-1)}\sum\sum sign(x_{i}-x_{j})\cdot sign\left( y_{i}-y_{j} \right)$$

Where $x_{i}$, $y_{i}$ are the ranks of the $i^{th}$ feature.

As we can observed from the formula, both Spearman and Kendall correlation focus on the ranks rather than the actual values. However, in the analysis of microbiome data, the significance of the actual abundance values of microbial taxa cannot be understated. Unlike Spearman’s and Kendall’s correlation methods, Pearson’s correlation evaluates the strength and direction of a linear relationship between two variables based on their actual values. This feature of Pearson’s correlation is particularly crucial in microbiome research, where the actual abundance of microbial taxa is a key factor driving the biological insights. Moreover, microbiome datasets often represent relative abundances, a characteristic that can lead to the occurrence of tied data. Tied data arise when multiple data points share the same value, a scenario not uncommon in microbiome studies due to the detection limits and the nature of relative abundance data. In statistical terms, ties refer to instances where two or more data points are assigned the same rank. The presence of a substantial number of ties can adversely affect the accuracy of rank-based correlation methods like Spearman’s and Kendall’s. This is because the calculation of these correlations relies on the assumption of distinct ranks for each data point, an assumption that is violated in the presence of ties. Consequently, tied data can diminish the precision of Spearman and Kendall correlations, potentially leading to less reliable estimates of association in microbiome datasets.

**Cutoff threshold validation**

To validate the optimal multiplier for the maximum cutoff approach, we systematically tested five different threshold multipliers (0.8X, 0.9X, 1.0X, 1.1X, and 1.2X) across three IBD datasets. The results consistently demonstrate that the 1.0X multiplier (maximum between-class correlation) provides the optimal balance for doppelganger identification.

| **Dataset** | **0.8X Cutoff** | **0.9X Cutoff** | **1.0X Cutoff** | **1.1X Cutoff** | **1.2X Cutoff** |
| --- | --- | --- | --- | --- | --- |
| **Dataset #1** | 90 pairs (3.7%) | 32 pairs (1.3%) | **9 pairs (0.4%)** | 1 pair (0.0%) | 0 pairs (0.0%) |
| **Dataset #2** | 53 pairs (2.2%) | 21 pairs (0.9%) | **10 pairs (0.4%)** | 0 pairs (0.0%) | 0 pairs (0.0%) |
| **Dataset #3** | 58 pairs (2.4%) | 33 pairs (1.3%) | **16 pairs (0.7%)** | 5 pairs (0.2%) | 0 pairs (0.0%) |

The 1.0X multiplier consistently identified a focused subset of highly similar pairs across all datasets, detecting 9-16 doppelganger pairs representing 0.4-0.7% of within-class sample pairs. In contrast, lower multipliers (0.8X and 0.9X) demonstrated excessive sensitivity, identifying 21-90 pairs (0.9-3.7% of within-class pairs), suggesting the inclusion of biologically plausible correlations that do not represent genuine anomalies. Conversely, higher multipliers (1.1X and 1.2X) showed severely reduced sensitivity, identifying 0-5 pairs (0.0-0.2% of within-class pairs), indicating potential loss of genuine doppelganger cases.

To further assess the effect of each cutoff on inference robustness, we performed bootstrap resampling (n = 100) to evaluate the variance of log2 fold changes between groups for each taxon. This analysis revealed that the 1.0X threshold consistently achieved a favorable balance—substantially reducing LFC variance compared to 0.8X and 0.9X, while maintaining sensitivity to true doppelgängers. Although slightly lower variance was sometimes observed at larger cutoff, the minimal number of removed samples suggests potential under-correction. Overall, the 1.0X multiplier offers the most reliable trade-off between effect size stability and comprehensive doppelgänger detection (Supplementary Figure 3).

**Removal of doppelgänger samples reveals additional biologically meaningful CRC-associated genera**

To assess the impact of doppelgänger removal on biological signal detection, we re-analyzed genus-level differential abundance in the Zeller cohort before and after filtering out identified doppelgänger pairs. As shown in the volcano plots, the core CRC-associated genera (*Fusobacterium, Parvimonas, Porphyromonas, Peptostreptococcus*) remained significant in both analyses, reflecting robust microbial signatures of disease. Notably, after doppelgänger removal, two additional genera—*Blautia* and *Bilophila*—emerged as statistically significant (padj < 0.05). *Blautia*, a well-established short-chain fatty acid-producing genus typically depleted in CRC, was only detected after filtering. Conversely, *Bilophila*, which has been associated with pro-inflammatory states and CRC enrichment, also became significant only after doppelgänger removal. These results suggest that eliminating overly similar sample pairs enhances the sensitivity of downstream analyses and enables more biologically meaningful discoveries.

**Expanded Model Performance Analysis**

To further investigate the effect of doppelgänger leakage, we extended the analysis to multiple machine learning models—K-nearest neighbors (KNN), Random Forest (RF), and Support Vector Machine (SVM)—and evaluated performance using accuracy, area under the curve (AUC), and F1 score (Supplementary figure 1). Model performance was assessed across progressive leakage scenarios, each repeated 20 times with different train/test splits. Boxplots in Supplementary Figure 1a show the distribution of results across repeats, capturing variability from random pair selection and split composition. Overall, performance tended to increase with leakage, with inflation observed at few points. The non-monotonic increases are attributable to several factors, including data quality, the nature of each ML approach, model parameterization, and the distribution of doppelgänger pairs within training and test sets.


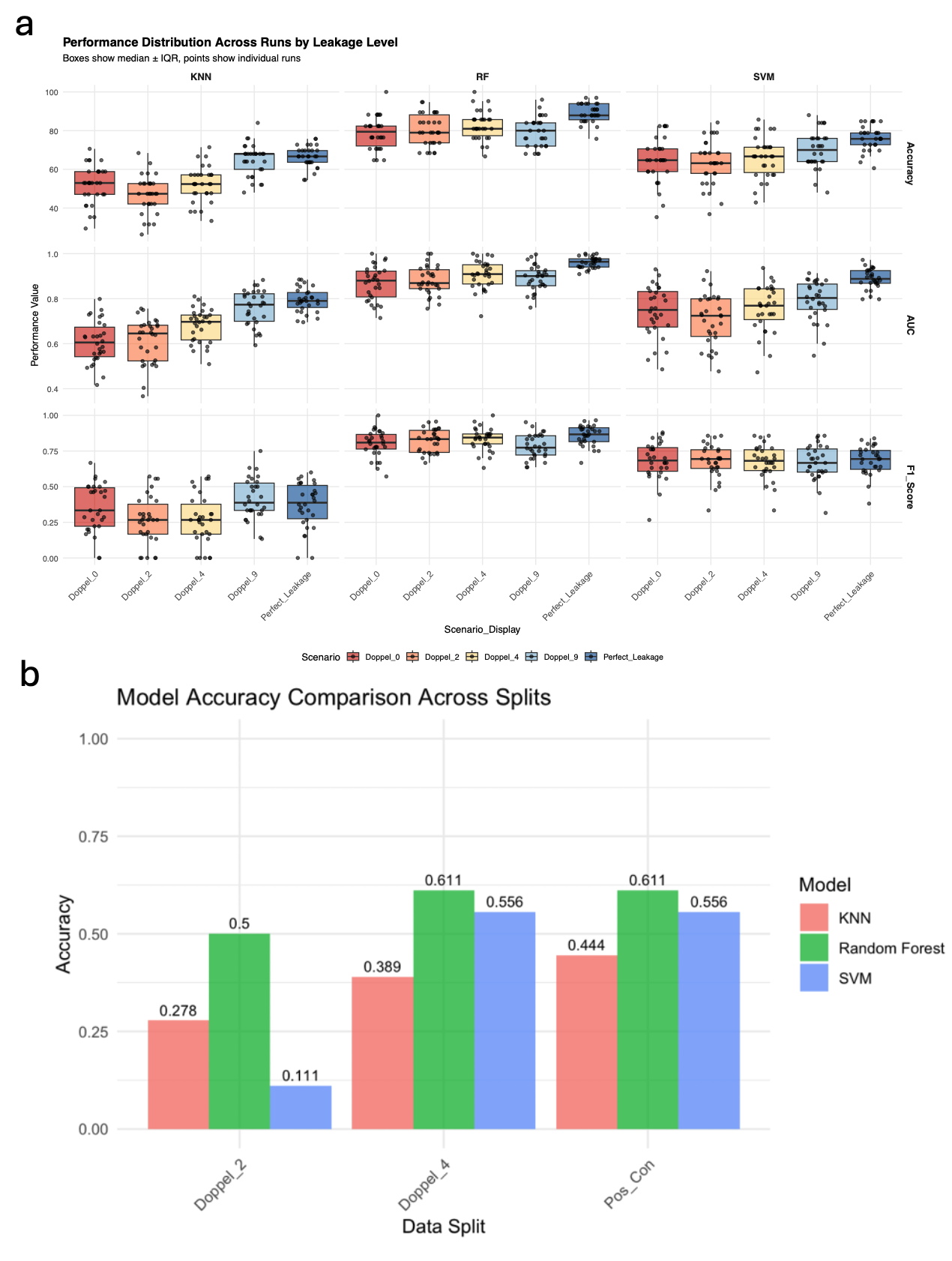


Supplementary figure 1: (a) Internal validation of metagenomics data results showing model performance across progressive doppelgänger leakage scenarios, repeated 20 times with different train/test splits. Scenarios include: No Leakage (0 pairs leaked to validation), progressive leakage (1, 2, 3, 4... pairs leaked to validation set), Max Leakage (all pairs leaked), and Perfect Leakage (identical pairs in both sets). Three metrics are reported: accuracy, area under the curve (AUC), and F1 score, across three classifiers: K-nearest neighbors (KNN), Random Forest (RF), and Support Vector Machine (SVM).

(b) Comparison of accuracy across KNN, Random Forest, and SVM under three representative split settings (Doppel_2, Doppel_4, and a positive control with maximum duplication). The trend confirms that inflated accuracy is not specific to any one model and persists across classifiers, further supporting the systematic nature of the doppelgänger effect.

Supplementary figure 2:

(a) Volcano plot of differential abundance analysis using the full dataset.

(b) Volcano plot after doppelgänger removal. Notably, *Blautia* (CRC-depleted) and *Bilophila* (CRC-enriched) became significant only after filtering, highlighting improved biological relevance.


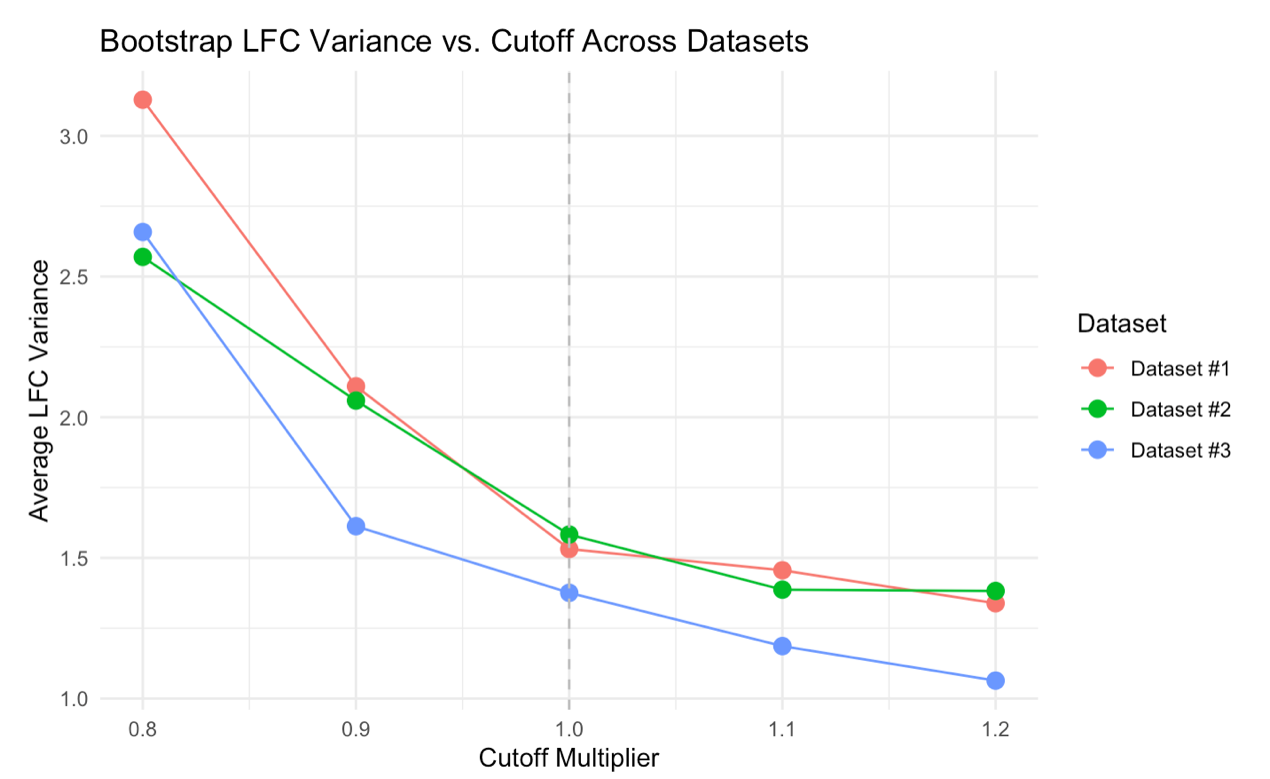


Supplementary figure 3: Bootstrap stability of log2 fold change (LFC) estimates across different doppelgänger cutoff thresholds.
